# Public perceptions and discussions of synthetic nicotine on Twitter

**DOI:** 10.3389/fpubh.2024.1370076

**Published:** 2024-07-26

**Authors:** Jiarui Chen, Jinxi He, Zidian Xie, Dongmei Li

**Affiliations:** ^1^Goergen Institute for Data Science, University of Rochester, Rochester, NY, United States; ^2^Department of Computer Science, University of Rochester, Rochester, NY, United States; ^3^Department of Clinical and Translational Research, University of Rochester Medical Center, Rochester, NY, United States

**Keywords:** synthetic nicotine, Twitter/X, public perception, health risks, nicotine addiction

## Abstract

**Background:**

As alternative replacement products for tobacco-derived nicotine, synthetic nicotine products have recently emerged and gained increasing popularity. This study analyzes public perception and discussion of synthetic nicotine products on Twitter (now “X”).

**Methods:**

Through Twitter streaming API (Application Programming Interface), we have collected 2,764 Twitter posts related to synthetic nicotine from December 12, 2021, to October 17, 2022, using keywords related to synthetic nicotine. By applying an inductive approach, two research assistants manually determined the relevance of tweets to synthetic nicotine products and assessed the attitude of tweets as positive, negative, and neutral of tweets toward synthetic nicotine, and the main topics.

**Results:**

Among 1,007 tweets related to synthetic nicotine products, the proportion of negative tweets (383/1007, 38.03%) toward synthetic nicotine products was significantly higher than that of positive tweets (218/1007, 21.65%) with a p-value <0.05. Among negative tweets, major topics include the concern about addiction and health risks of synthetic nicotine products (44.91%) and synthetic nicotine as a policy loophole (31.85%). Among positive tweets, top topics include alternative replacement for nicotine (39.91%) and reduced health risks (31.19%).

**Conclusion:**

There are mixed attitudes toward synthetic nicotine products on Twitter, resulting from different perspectives. Future research could incorporate demographic information to understand the attitudes of various population groups.

## 1 Introduction

According to the World Health Organization (WHO), over 1 billion people globally are current tobacco smokers (1). The U.S. Food and Drug Administration (FDA) recognizes tobacco use as a significant preventable cause of disease and death in the United States, with approximately 28.3 million adults currently smoking cigarettes (2). The adverse health effects of smoking, ranging from respiratory complications to cardiovascular diseases and cancer, are well-established. Smoking has been linked to higher rates of diabetes, chronic kidney disease, and increased postoperative complications in cancer patients (3, 4). The FDA has implemented stringent regulations on nicotine-containing products to combat this, including restricting marketing and sales to minors, mandatory warning labels for smokeless tobacco, and required disclosure of ingredients (5). However, as traditional smoking rates have declined in specific demographics, vaping or using electronic cigarettes (e-cigarettes) emerged as a new trend, especially among the younger generation (6). This rise in vaping has prompted numerous studies to characterize the prevalence, risk factors, and associated health complications in different countries (7, 8). Vaping is associated with physical and behavioral health risks, emphasizing the importance of informing pediatricians and clinicians about these risks to provide quality patient care (9). The FDA’s regulatory approach to e-cigarettes and vaping devices is multifaced. It monitors usage rates, particularly among youth, and has launched educational campaigns to deter initiation; it regulates the manufacture, import, packaging, labeling, advertising, promotion, sale, and distribution of Electronic Nicotine Delivery Systems (ENDS) and their components which includes e-liquids, vials that contain e-liquid, cartridges, flavors, certain batteries, and even software (10).

Central to both smoking and vaping is nicotine, a highly addictive substance. Its addictive nature is one of the primary reasons most individuals find it challenging to quit smoking or vaping once they start, and it is responsible for the widespread use of tobacco products and the pronounced withdrawal effects that arise upon cessation, making it difficult for individuals to quit (11). However, to stay ahead of regulatory curves, the ever-evolving tobacco industry has introduced synthetic nicotine products (12). Except for containing no tobacco or its impurities, this laboratory-produced compound is chemically identical to tobacco-derived nicotine (13). This distinction has led to a regulatory gray area, as products containing synthetic nicotine are not presently classified as tobacco products. Consequently, the FDA needs to be more transparent concerning its oversight. Capitalizing on regulatory uncertainty, many e-cigarette companies have shifted to producing tobacco-free nicotine (TFN) products. This move allows them to bypass the FDA regulations. These products include disposable vape pens like Puff Bar and Elf Bar, nicotine pouches such as Zyn and On!, and nicotine-infused toothpicks from brands like Pixotine and Zippix. As of April 2022, synthetic nicotine starts to be regulated by the FDA due to its addictive properties. This regulation ensures that products with tobacco-free nicotine comply with the Federal Food, Drug, and Cosmetic Act. This Act prohibits the sale of such products to individuals under 21 years old and bans the distribution of free samples. Additionally, it requires manufacturers to register with the FDA and submit a premarket application for authorization (14).

As the common platform for the public to share their opinions and experiences, social media can be used to disseminate health-related information, investigate policy influence, and collect opinions among the general public. As contemporary public health challenges such as the vaping epidemic in youth evolve, social media like Twitter/X allows researchers and policymakers to engage directly with the public, monitor reactions to health news and policy changes, and foster community discussions (15, 16). As of December 2022, Twitter/X has over 368 million monthly active users worldwide with vigorous most current and updated public testimonies and discussions (17). Social media data have been widely used in previous research to investigate the public perceptions and discussions of various tobacco products and the influence of tobacco product-related policies, including electronic cigarettes (e-cigarettes), snus, waterpipe tobacco products, oral nicotine pouches, heated tobacco products, and synthetic cooling agents used in e-cigarettes (18–23).

This study aims to explore and analyze the public perception of synthetic nicotine on Twitter, focusing on the attitudes and opinions expressed in tweets on synthetic nicotine products collected from December 12, 2021, to October 17, 2022, by analyzing Twitter data and identifying prevalent attitudes. We have selected this specific time period in our study for two reasons. Firstly, this period covers a series of changes in government legislation related to synthetic nicotine and e-cigarette products, which occurred between March 2022 and July 2022. Secondly, this time frame allows us to have adequate data before and after the legislative changes to make any potential comparisons. By doing so, we will be able to examine the potential impact of these changes on public attitudes toward synthetic nicotine and make meaningful comparisons. In terms of methodology, we utilized a combination of keyword filtering, data cleaning, and hand-coding to obtain a dataset of 2,764 tweets, which were further analyzed to categorize attitudes (positive, neutral, and negative) and identify the major topics of discussion. Our findings reveal the temporal popularity of synthetic nicotine-related content on Twitter and offer insights into the arguments for and against using these products. We discuss the potential impact of government regulations and announcements on public perception and compare our results with previous studies in the field.

## 2 Methods

### 2.1 Data collection and pre-processing

Using the Twitter streaming application programming interface (API), we collected Twitter data from December 12, 2021, to October 17, 2022, using keywords related to cigarettes and nicotine, including “cig*,” “smok*,” “nicotine,” and “tobacco.” Then, we filtered the data using a set of synthetic nicotine-related keywords, including “synthetic nicotine,” “synthetic,” “synthetic s-nicotine,” “tobacco-free nicotine,” “tobacco-free nicotine,” “tobacco-free nicotine,” “tfn,” “r-nicotine,” “non-tobacco-derived nicotine,” and “synthetic tobacco (24, 25).” Since this study focuses on public perception and discussion about synthetic nicotine products, we removed all commercial Twitter posts. First, if a tweet is posted by a user with a username containing “dealer,” “store,” “supply,” “promo,” etc., it is considered commercial and thus removed. In addition, a tweet is also considered commercial if its text includes words such as “sale,” “discount,” “percent off,” “wholesale,” etc. (26). We used the Python SequenceMatcher to remove highly similar tweets to refine the data processing steps. After removing duplicate tweets and retweets, we obtained a data set with 2,764 tweets related to synthetic nicotine.

### 2.2 Content analysis

We followed the inductive approach used in previous literature to manually code the tweets for our content analysis (27). To develop a codebook, we randomly sampled approximately 10% of the tweets (291 out of 2,764 tweets). First, each tweet was classified as either synthetic-nicotine-related, noise, or commercial (due to some misclassification using the filtering keywords), with the aim of excluding irrelevant and commercial tweets that did not contribute to the public perception and discussions of synthetic nicotine products. Next, we determined whether the tweet was about synthetic nicotine products or the related policy. In this study, we only considered product-related tweets, as we aimed to examine the public perceptions and discussions of synthetic nicotine products. Therefore, we excluded tweets related to synthetic nicotine policies. In total, we identified 1,007 tweets related to synthetic nicotine and related products. Then, following the previous attitude classification strategy (28), we grouped the tweets into three attitude categories (positive, neutral, and negative) according to the user’s attitude toward synthetic nicotine and related products. Finally, tweets within each attitude category were grouped into different topics based on the reasoning and argument presented in the tweets. As shown in Supplementary Table S1, there are four topics in positive tweets, including an alternative replacement for nicotine, reduced health risks, good taste, and others. Negative tweets have six main topics, including synthetic nicotine as a policy loophole, addiction, and health risks, not a good alternative, bad taste, no reason, and others. Tweets with a neutral attitude have two major topics: general discussion and comparison with regular nicotine. Two individual human coders labeled the sampled tweets, respectively. The inter-rater reliability between the two coders reached a kappa statistic of 0.88 for the attitude and a kappa statistic of 0.88 for the topics. Any discrepancies were discussed and reconciled among a group of four, including the two original coders. Finally, the two coders single-coded the remaining tweets based on the codebook (Supplementary Table S1).

## 3 Results

From December 12, 2021, to October 17, 2022, we have identified 1,007 tweets related to synthetic nicotine. Within the study period, we did not observe any obvious trend in discussing synthetic nicotine (Supplementary Figure S1). Among 1,007 tweets, 218 (21.65%) tweets showed a positive attitude, 406 (40.32%) tweets were neutral, and 383 (38.03%) tweets had a negative attitude. The proportion of negative tweets is significantly higher than that of the positive ones (*p* = 0.000036).

As shown in Table 1, among positive tweets, the top topics were “Alternative Replacement for Nicotine” (39.91%), followed by “Others” (31.19%) and “Reduced Health Risk” (27.52%). In the negative category, the most discussed topics were “Addiction and Health Risks” (44.91%), followed by “Synthetic Nicotine as a Policy Loophole” (31.85%) and “No Reason” (15.14%). Among both the positive topics and the negative topics, “Addiction and Health Risks” was the most prevalent one, and a further look into the topic showed that 63.95% (110 out of 172) of the tweets labeled mentioned teenagers or young adults indicated the wide-spread concern about synthetic nicotine’s health impact on younger user groups. In addition, it is noticeable that little attention was paid to the product’s taste for both positive and negative tweets, comprising only 1.38% and 1.57% of the discussion, respectively. Among tweets with a neutral attitude toward synthetic nicotine products, 382 (94.09%) of them are general mentions of synthetic nicotine and related products, followed by the topic of “comparison with regular nicotine” (24, 5.91%).

**Table 1:** Distribution of topic discussed within each attitude category.

Attitude (n, %)	Topic	Number of tweets, n (%)
Positive (218, 21.65%)	Alternative replacement for nicotine	87 (39.91%)
	Others	68 (31.19%)
	Reduced health risks	60 (27.52%)
	Good taste	3 (1.38%)
Negative (383, 38.03%)	Addiction and health risks	172 (44.91%)
	Synthetic nicotine as a policy loophole	122 (31.85%)
	No reason	58 (15.14%)
	Not a good alternative	19 (4.96%)
	Bad taste	6 (1.57%)
	Others	6 (1.57%)
Neutral (406, 40.32%)	General discussion	382 (94.09%)
	Comparison with regular nicotine	24 (5.91%)

Building on the earlier observations regarding regulatory changes and their impact on public perception and discussions, we delved into the sentiment shifts, specifically around introducing the new FDA policy regulating synthetic nicotine products in April 2022, focusing on the proportional change in tweets. Table 2 reveals nuanced shifts in public sentiment when we compare the proportions of tweets on different topics before and after the FDA policy on synthetic nicotine products. There was a notable decrease in the proportion of tweets about synthetic nicotine as a policy loophole (−9.59%) and addiction and health risks (−3.72%), suggesting that the public may perceive the FDA policy as effectively addressing these significant concerns. Two topics in the tweets with a positive attitude, alternative nicotine products (−2.67%) and good taste (−2.29%), had a decreased percentage after the FDA policy on synthetic nicotine products, hinting a reduced interest in these synthetic nicotine products. At the same time, there was a slight increase (2.91%) in the topic of “reduced health risks,” suggesting a slightly increased perception of synthetic nicotine products as health risk reduction. In addition, there was a marked increase in general discussions of synthetic nicotine products (8.49%), possibly reflecting more public conversations about synthetic nicotine products after the FDA policy.

**Table 2:** Impact of the FDA policy regulating synthetic nicotine on public perception of synthetic nicotine products.

Attitude	Topic	Change in proportion (After vs. Before the FDA policy)
Positive	Alternative replacement for nicotine	−2.67%
	Others	1.31%
	Reduced health risks	2.91%
	Good taste	−2.29%
Negative	Addiction and health risks	−3.72%
	Synthetic nicotine as a policy loophole	−9.59%
	No reason	4.19%
	Not a good alternative	0%
	Bad taste	0%
	Others	0.34%
Neutral	General discussion	8.49%
	Comparison with regular nicotine	0.62%

## 4 Discussion

Our study showed that negative tweets about synthetic nicotine dominated Twitter from December 12, 2021, to October 17, 2022. The negative attitudes toward synthetic nicotine mainly focused on the loophole nature of synthetic nicotine products and the concerns about teenagers’ usage of the products. On the other hand, reasons for the positive attitude include synthetic nicotine being an alternative to help quit smoking and its harm reduction property.

Regarding whether synthetic nicotine is an alternative to tobacco-derived nicotine, we found that more tweets agreed that synthetic nicotine is a good alternative to regular tobacco-derived nicotine products than those who disagreed (87 vs. 19). This corresponds to the promotion strategy of synthetic nicotine products. As pointed out in a white paper about the marketing of synthetic nicotine products, they are marketed as alternatives to traditional tobacco by being portrayed as irrelevant to the public’s stereotypical impression of traditional tobacco (29). This could be the reason for the dominating number of tweets about synthetic nicotine being an alternative spotted in our study. According to a recent study, the use of the term “tobacco-free” associated with synthetic nicotine products may make people perceive them as less risky and more attractive (8). As a result, there has been a significant increase in the number of tweets advocating for synthetic nicotine as a safe alternative.

Despite the discussion on synthetic nicotine being the alternative, we noticed that the most discussed topic among positive and negative tweets was the addiction and health risks of the products (Table 2). Previous studies on the health effects of synthetic nicotine products backed up the legitimacy of these discussions. For example, a study showed that a non-tobacco-based product increases nicotine uptake in blood circulation (30). Further, the harmful effects of nicotine discussed in the tweets, such as the increased risk of respiratory disorders and harm to the immune system, were also reported in previous literature (31). A recent study conducted in the New England region found that 62.29% of the participating adolescents and 64.13% of the adults perceived tobacco-free nicotine as equally or more addictive than tobacco-derived nicotine (24). These results might contribute to the negative attitudes and the discussion surrounding addiction and health effects of synthetic nicotine. Meanwhile, we noticed that the discussion about synthetic nicotine being a regulatory loophole was also popular, and we speculated that this was triggered by media articles with similar arguments (32). While there were accusations pointing out synthetic nicotine and tobacco-free nicotine products escaped from regulation, this loophole was closed by the FDA as of April 2022 (14).

We explored the fluctuations in public attitudes toward synthetic nicotine products over four peaks, specifically focusing on positive, negative, and neutral sentiment changes (Supplementary Figure S1 and Supplementary Table S2). By linking these shifts in sentiment to news events and policy changes, we hope to understand better how external factors can influence the public’s perception of synthetic nicotine products. Through examining the data from March to July 2022, we identified significant variations in attitudes, which can be attributed to developments such as the FDA gaining new powers, banning certain synthetic nicotine products, and placing an administrative hold on a marketing denial order (Supplementary Figure S1). The attitudes toward synthetic nicotine products have fluctuated significantly, with a surge in negative sentiment in March 2022, followed by a gradual decline in negativity and an increase in neutrality. This change in sentiment can be attributed to various news and regulatory developments. In March 2022, Congress moved to grant the FDA new powers over synthetic nicotine products, including the popular Puff Bar e-cigarettes. This decision, reported by the Washington Post, likely contributed to the spike in negative attitudes as the regulation aimed to curb youth consumption of synthetic nicotine products (33). By April 2022, the new law became effective, closing the synthetic nicotine loophole and intending to reduce nicotine addiction among US teens using flavored e-cigarettes. This development, reported by JAMA Network, may have led to a decrease in negative sentiment and an increase in neutral opinions as the regulation took effect (34). In June 2022, the FDA issued marketing denial orders (MDOs) to ban Juul products from being sold in the US (35). This decision could have influenced the positive sentiment to rise slightly, as it was another measure to protect young people from e-cigarette addiction. However, the FDA later placed an administrative hold on the ban to review Juul’s marketing application again, which may have contributed to a further decline in negative sentiment and an increase in neutral opinions in July 2022.

There are several limitations in our study. While we tried to be as inclusive as possible when collecting synthetic-nicotine-related keywords, the keyword list we employed during data collection may not be comprehensive enough to include all relative contents on Twitter. In addition, we did not remove social bots in this study, which might introduce some biases. Moreover, due to the quick evolving of tobacco and nicotine products, our findings may not accurately reflect the current trend and status quo, which need to be updated in the future study. While basic demographics of Twitter users are not available, we could not determine the attitudes between different demographic groups. Finally, Twitter users could not fully represent the whole population.

## 5 Conclusion

This study showed public attitudes and opinions toward synthetic nicotine on Twitter. Our analysis showed that negative attitudes outweighed positive ones on Twitter during the span of the study. Helping smokers quit traditional tobacco products was the leading topic in favor of synthetic nicotine products. On the other hand, opposing voices prevalently argued that it was a regulatory loophole. Being an alternative to help quit smoking traditional tobacco products and being a regulation loophole were the leading arguments in favor and against synthetic nicotine products, respectively. The study further showed how regulation changes and announcements could potentially shift the attitude distribution toward the product. This study provided valuable information about the user perception of synthetic nicotine on Twitter and could help gaining knowledge about product consumption behaviors. The perceived reduced risk of synthetic nicotine on social media (such as Twitter/X) prompts us to advocate for further clinical studies on the health effects of electronic cigarettes containing synthetic nicotine, and further convey such information on the Internet to educate the public (36).

## Data availability statement

Publicly available datasets were analyzed in this study. This data can be found at: https://twitter.com.

## Ethics statement

This study has been reviewed and approved by the Office for Human Subject Protection Research Subjects Review Board (RSRB) at the University of Rochester (Study ID: STUDY00006570). The studies were conducted in accordance with the local legislation and institutional requirements. Written informed consent for participation was not required from the participants or the participants’ legal guardians/next of kin in accordance with the national legislation and institutional requirements.

## Author contributions

JC: Formal analysis, Investigation, Validation, Visualization, Writing – original draft, Writing – review & editing. JH: Formal analysis, Investigation, Validation, Visualization, Writing – original draft, Writing – review & editing. ZX: Conceptualization, Data curation, Investigation, Methodology, Project administration, Resources, Supervision, Writing – original draft, Writing – review & editing. DL: Conceptualization, Funding acquisition, Investigation, Project administration, Supervision, Writing – original draft, Writing – review & editing.
