# Supplementary material for: Public perceptions and discussions of synthetic nicotine on Twitter
Source: Front Public Health. 2024 Jul 26;12:1370076. doi: 10.3389/fpubh.2024.1370076 (PMC11310114; doi:10.3389/fpubh.2024.1370076)
Supplement: Supplementary file 1 [file Data_Sheet_1.docx]

Supplementary Material

**Supplemental Table S1. Codebook for hand-coding synthetic nicotine-related tweets.**

| **Attitude** | **Topic** | **Description** | **Example** |
| --- | --- | --- | --- |
| Positive | Alternative replacement for nicotine | Synthetic nicotine is a useful replacement for regular tobacco products and helps quit smoking. | “tobacco harm reduction products just took a huge hit when you signed that bill. synthetic nicotine is helping thousands stay off deadly cigarettes that cause 30% of the cancer in america. youth nicotine vaping is very low and youth smoking is miniscule.” |
|  | Others | The tweet expresses no specific reasons for favoring synthetic nicotine but shows a positive attitude in general. | “if nobody got me i know that synthetic nicotine products got me.” |
|  | Reduced Health Risks | The tweet focuses on the harm reduction property of synthetic nicotine. | “yes if you stay with pure synthetic nicotine your risks are fairly low, not zero, but lower than almost all other e-cigs.  however, almost all e-cigs that younger adults and teens use aren't just pure synthetic nicotine, they definitely are fairly harmful.” |
|  | Good Taste | Synthetic nicotine products taste good. | “this. i also found out there‚Äôs synthetic nicotine??? now idk how bad that is but damn it tastes good asf” |
| Negative | Synthetic Nicotine as a Policy Loophole | The tweet claims that synthetic nicotine is a loophole evading government regulation. | “sales are rising of flavored e-cigarettes using synthetic nicotine that evades regulatory oversight, a gap that lawmakers are now trying to close.” |
|  | Addiction and Health Risks | The tweet focuses on the potential harm that synthetic nicotine could impose on the youth. | “#synthetic #nicotine comes from a #laboratory rather than #tobacco . but regardless of source, nicotine is an addictive #drug with known harms for young people.  learn more about synthetic nicotine in nicotine pouches and e-cigarettes” |
|  | No reason | The tweet expresses no specific reasons for opposing synthetic nicotine but shows a negative attitude in general. | “we want synthetic nicotine products to disappear from the market.” |
|  | Not a Good Alternative | The tweet claims that synthetic nicotine is not a replacement for traditional tobacco products and does not help quit smoking. | “i vaped to quit smoking. they banned nicotine from all vape juice and replaced it with synthetic nicotine. not the same. doesn’t work. now i’m smoking again” |
|  | Bad Taste | The tweet points out that synthetic nicotine products taste bad. | “synthetic nicotine is the worst. it fills your receptors, but gives no satisfaction.” |
|  | Others | Other tweets with negative attitudes that do not belong to any categories above | “china wins again! yet the fda will let china keep selling synthetic nicotine to americans! make it make since. 80% ofvape market is made up of synthetic nicotine made by china. yet let'skill an american company and it's employees that will lose livelihood and insurance for their kids.” |
| Neutral | General Discussion | The tweet is a general discussion about synthetic nicotine with no obvious attitudes. | “synthetic nicotine is produced in a lab. if it was extracted from tobacco it is not synthetic nicotine. just like a synthetic beef burger is produced in a lab, and is not made of beef.” |
|  | Comparison with Regular Nicotine | The tweet highlights the similarity or difference between synthetic nicotine and regular tobacco. | “lots of e-cigarettes contain 100% synthetic nicotine. the same nicotine in nicorette gum, patches, lozenges, and sprays. that nicotine is not related to tobacco at all. nicotine also occurs naturally in other plants as well, like eggplant and tomato plants. are they tobacco too?” |

**Supplemental Table S2. Attitudes towards synthetic nicotine products in months containing peak.**

| **Date** | **Sentiment** | **Number of Tweets (%)** |
| --- | --- | --- |
| March, 2022 | Positive | 93 (27.8%) |
|  | Negative | 120 (35.8%) |
|  | Neutral | 122 (36.4%) |
| April, 2022 | Positive | 22 (16.4%) |
|  | Negative | 33 (24.6%) |
|  | Neutral | 79 (59.0%) |
| June, 2022 | Positive | 29 (26.9%) |
|  | Negative | 30 (27.8%) |
|  | Neutral | 49 (45.3%) |
| July, 2022 | Positive | 33 (23.2%) |
|  | Negative | 45 (31.7%) |
|  | Neutral | 64 (45.1%) |


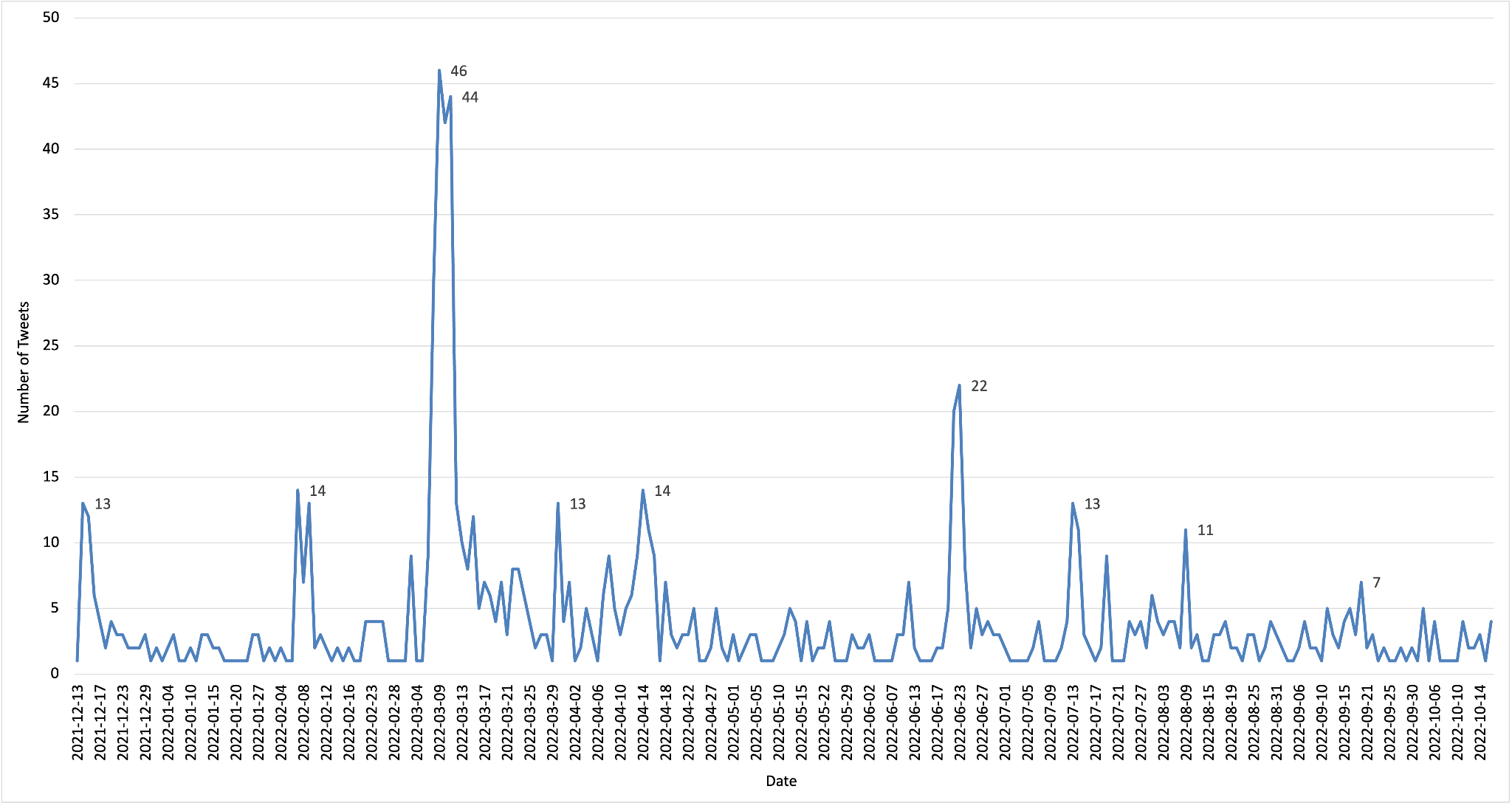


**Supplemental Figure S1. Temporal trend of Tweets related to synthetic nicotine.**
